# L-Cysteine as an Irreversible Inhibitor of the Peroxidase-Mimic Catalytic Activity of 2-Dimensional Ni-Based Nanozymes

**DOI:** 10.3390/nano11051285

**Published:** 2021-05-13

**Authors:** Piyumi Dinusha Liyanage, Pabudi Weerathunge, Mandeep Singh, Vipul Bansal, Rajesh Ramanathan

**Affiliations:** Ian Potter NanoBioSensing Facility, NanoBiotechnology Research Laboratory (NBRL), School of Science, RMIT University, GPO Box 2476, Melbourne, VIC 3000, Australia; s3437386@student.rmit.edu.au (P.D.L.); pabudi.weerathunge@rmit.edu.au (P.W.); mandeep.singh@rmit.edu.au (M.S.)

**Keywords:** nanozyme, L-cysteine, inhibitor, irreversible inhibitor, enzyme-mimic, peroxidase-mimic

## Abstract

The ability to modulate the catalytic activity of inorganic nanozymes is of high interest. In particular, understanding the interactions of inhibitor molecules with nanozymes can bring them one step closer to the natural enzymes and has thus started to attract intense interest. To date, a few reversible inhibitors of the nanozyme activity have been reported. However, there are no reports of irreversible inhibitor molecules that can permanently inhibit the activity of nanozymes. In the current work, we show the ability of L-cysteine to act as an irreversible inhibitor to permanently block the nanozyme activity of 2-dimensional (2D) NiO nanosheets. Determination of the steady state kinetic parameters allowed us to obtain mechanistic insights into the catalytic inhibition process. Further, based on the irreversible catalytic inhibition capability of L-cysteine, we demonstrate a highly specific sensor for the detection of this biologically important molecule.

## 1. Introduction

Nanozymes are inorganic nanomaterials that are believed to mimic the catalytic activity of natural enzymes [1,2,3,4,5,6,7,8,9,10,11,12]. To date, a range of metal, metal-oxide, metal sulfide and carbon-based nanozymes have been reported for their enzyme-mimicking catalytic activity. This activity has been employed for a range of applications ranging from controlling the bacterial growth, prodrug therapy, and photodynamic therapy to sensing of analytes [1,2,3,4,5,6,7,8,9,10,11,12]. A recent review reported that there are over 200 laboratories globally working on developing new nanozymes and expanding their applicability [13]. Of the different applications and fundamental investigations, one aspect that is gaining rapid attention is the ability to modulate the catalytic activity of nanozymes i.e., either enhance, deteriorate, temporarily inhibit or permanently inhibit its catalytic activity. To this end, metal ions [14,15,16,17], biomolecules such as ATP [18,19,20], cysteine [21,22,23,24,25], and glutathione [26,27] have all been successfully employed to modulate the catalytic activity of nanozymes. For instance, ATP was seen to enhance the catalytic activity of nanozymes while the amino acid cysteine was reported to inhibit their catalytic activity [18,21]. In this context, inhibition of the nanozyme activity can be considered akin to natural enzymes where an inhibitor molecule binds either to the active site of the enzyme or away from the active site of the enzyme resulting in complete loss of the catalytic activity or lowering the catalytic efficiency [28]. The process of inhibition can either be temporary or permanent. In the case of temporary inhibition, the inhibitor molecule binds reversibly to an enzyme (non-covalent interaction) such that it either temporarily inhibits or slows down the catalytic rate. In contrast, irreversible inhibitors bind covalently to the enzyme and permanently block its activity [29]. It is important to note that in the context of nanozymes, the only type of inhibition reported to date is reversible inhibition [30,31]. Further, in reversible inhibition, calculating the steady state kinetic parameters viz. Michaelis constant (*K_m_*) and maximum velocity of reaction (*V_max_*) have offered deep insights into the mechanism of reversible inhibition (competitive vs. uncompetitive vs. non-competitive) [18]. Although such understanding has allowed the development of new inhibitor molecules for inorganic nanozymes, irreversible inhibitors of nanozymes have not been reported so far, at least to the best of the authors’ knowledge. 

Our group and others have previously established that L-cysteine can act as a nanozyme inhibitor, thereby inactivating the peroxidase-mimic catalytic activity resulting in a turn-off nanozyme sensor [21,22,24,32]. Most of these studies suggested that L-cysteine shields the surface of the nanozyme from the colorimetric substrate resulting in the loss of catalytic activity [22,32]. Our group was the first to show that in the case of Gd(OH)_3_ nanozyme, L-cysteine acts as a competitive inhibitor molecule for the nanozyme substrate which results in temporary inactivation of the catalytic activity [21]. While the investigation of the underlying mechanism has provided some understanding of the complex molecular interactions, it is still unclear if most nanozymes will reversibly interact with L-cysteine and if L-cysteine interacts reversibly, what is the mechanism of this reversible inhibition? Also, what parameters dictate the reversible interaction? 

In the current study, we assess the mode of molecular interaction of L-cysteine with Ni-based nanozymes. For this, we developed a simple solution-based route to fabricate *β*-Ni(OH)_2_ and NiO nanomaterials. The catalytic enzyme-mimicking behaviour of both materials was established where the oxide phase showed superior peroxidase-mimic catalytic activity than the hydroxide phase. Exposure of the NiO nanoparticles to L-cysteine resulted in permanent inhibition of the catalytic activity where the degree of inhibition was dependent on the concentration of L-cysteine. Unlike natural enzymes where one inhibitor molecule typically results in the inactivation of a single enzyme molecule by interacting at the active site; in the case of nanozymes, the surface atoms of the nanoparticles play the equivalent role of the enzyme active sites [31]. The exposure of NiO to low concentrations of L-cysteine results only in partial loss of its activity as the surface of NiO is not blocked completely. In contrast, higher concentrations of L-cysteine result in complete loss of its catalytic activity. Unlike our previous work on Gd(OH)_3_ nanozyme, the nanozyme activity of NiO does not regain with time at a fixed L-cysteine concentration. Based on the enzyme kinetic theory, we observed that the affinity of the nanozyme to its substrate (*K_m_*) remained unchanged while the catalytic efficiency decreased. This suggests that more than one L-cysteine molecule is bound to the surface of each NiO blocking the surface atoms. Interestingly, other amino acids did not influence the activity of the NiO nanozyme showing high specificity of L-cysteine to bind to the NiO nanozyme. FTIR analysis further confirmed the presence of L-cysteine on the surface of the NiO nanozyme. Based on the high specificity of interactions between L-cysteine and NiO nanozyme, we further exploited this system to develop a simple L-cysteine sensor where the system allowed us to detect L-cysteine even in the presence of other amino acids and other sulfur containing compounds as contaminants either independently or in a mixture.

## 2. Materials and Methods

### 2.1. Materials and Reagents

Nickel(II) chloride, triethylamine, all peroxidase substrates and amino acids were purchased from Sigma Aldrich (Australia) and hydrogen peroxide was purchased from Chem Supply (Australia). All chemicals were used as received.

### 2.2. Synthesis of β-Ni(OH)_2_ and NiO

A nickel precursor salt solution (40 mL of 1 M NiCl_2_) was first added to a round-bottom flask and heated to 80 °C in an oil bath under continuous stirring where the temperature was continuously monitored using a thermometer. Once the desired temperature was achieved, triethylamine (5 mL) was introduced under continuous stirring. The reaction was allowed to stir for 1 h at a constant temperature of 80 °C. The resultant precipitate was allowed to cool and washed thoroughly using a mixture of acetone: methanol (1:1 *v*/*v*) followed by vacuum drying. The greenish powder obtained contained the *β*-Ni(OH)_2_ nanomaterial. For the synthesis of the NiO, the greenish powder of *β*-Ni(OH)_2_ was calcined at 400 °C for 12 h in the air to obtain a black powder of NiO.

### 2.3. Material Characterization

Both nanomaterials were thoroughly characterized using a suite of materials characterization tools including transmission electron microscopy (TEM) and high resolution TEM (HR-TEM), energy dispersive X-ray (EDX) spectroscopy, X-Ray diffraction (XRD), atomic emission spectrometer (AES), Raman spectroscopy and Zetasizer. A detailed characterization is given in the Supplementary Information.

### 2.4. Peroxidase-Mimic Catalytic Activity of Nanozymes and Optimization 

The catalytic performance of both nanozymes was first assessed by exposing a fixed concentration of the nanozyme (the equivalent of 1.5 mM Ni ion concentration) to 0.5 mM 2,2′-azino-bis(3-ethylbenzothiazoline-6-sulfonic acid) diammonium salt (ABTS), 3,3’,5,5’-tetramethylbenzidine (TMB) and *o*-phenylenediamine dihydrochloride (OPD) substrates in the presence and absence of 10 mM H_2_O_2_ in glycine-HCl buffer at pH 3 and 30 °C. The change in the color of the substrates was monitored at 405 nm, 650 nm and 450 nm, respectively, for all the three substrates, using a multimode plate reader. The change of absorbance (ΔA) was calculated by subtracting the absorbance value of the pristine nanozyme (background used as A_0_) from the absorbance at time point *t* (*A_t_*). The total volume of all reactions was maintained at 200 µL. To understand if the catalytic activity is an inherent property of the nanozyme, both nanozymes were independently exposed to acidic buffer (pH 3) for 30 min. The nanozymes were then centrifuged and the leached Ni ion concentration in the supernatant was determined by AES. Subsequently, the leached Ni ions from this experiment were also evaluated as a catalyst. Additionally, we also performed control experiments where known concentration of Ni^2+^ ions were used as the catalyst. The optimization of catalytic activity was assessed by changing the concentration of nanozyme (the equivalent of Ni ions from 0–1.75 mM), pH (1–8) and reaction temperature (10–80 °C) while keeping a fixed concentration of ABTS (0.5 mM) and H_2_O_2_ (10 mM). The steady state kinetic parameters were calculated by performing experiments by varying either the ABTS (0–8 mM) or H_2_O_2_ concentration (0–200 mM) at a specific time point under optimum reaction conditions (pH 3, Ni concentration 1.5 mM and temperature 30 °C). The absolute reaction velocity was calculated using Beer-Lambert Law wherein the absorbance value of oxidised ABTS was converted into product concentration using the molar extinction coefficient of oxidised ABTS (36,000 M^−1^·cm^−1^). The Michaelis constant (*K_m_*) and initial maximum velocity of the reaction (*V_max_*) were calculated using non-linear curve fitting in OriginPro 2016 (OriginLab, Northampton, MA, USA).

### 2.5. Interaction of L-Cysteine Amino Acid with NiO Nanozyme

The NiO nanozyme was first exposed to three different concentrations of L-cysteine (10, 100 and 200 µM) for 10 min. Following this, the substrates ABTS and H_2_O_2_ were added to the reaction and the inhibition of the catalytic performance was measured as a function of time. The ABTS (0.5 mM), H_2_O_2_ (10 mM) and Ni concentration (1.5 mM) were kept constant during these experiments. The nanozyme was also exposed to other commonly occurring amino acids and several thiolic compounds (cystine, cysteamine, thiourea, sodium 2-propanthiolate, and homocysteine) at a fixed concentration of 200 µM. The potential inhibition of the catalytic activity was calculated after 10 min of incubation with ABTS and H_2_O_2_ (the protocol was similar to the initial assay). A systematic concentration-dependent study was further performed where the NiO nanozyme was exposed to increasing concentrations of L-cysteine (0–500 µM) keeping all other steps consistent. In another experiment, we exposed a fixed concentration of L-cysteine to H_2_O_2_ for 10 min followed by the addition of ABTS and NiO nanozyme. The linear regression of the change in absorbance was determined using OriginPro 2016. All sensor parameters were calculated using methods employed in our previous work [33]. The *K_m_* and *V_max_* were also calculated in the presence of two different L-cysteine concentrations (25 µM and 50 µM).

## 3. Results and Discussion

**Figure 1:**
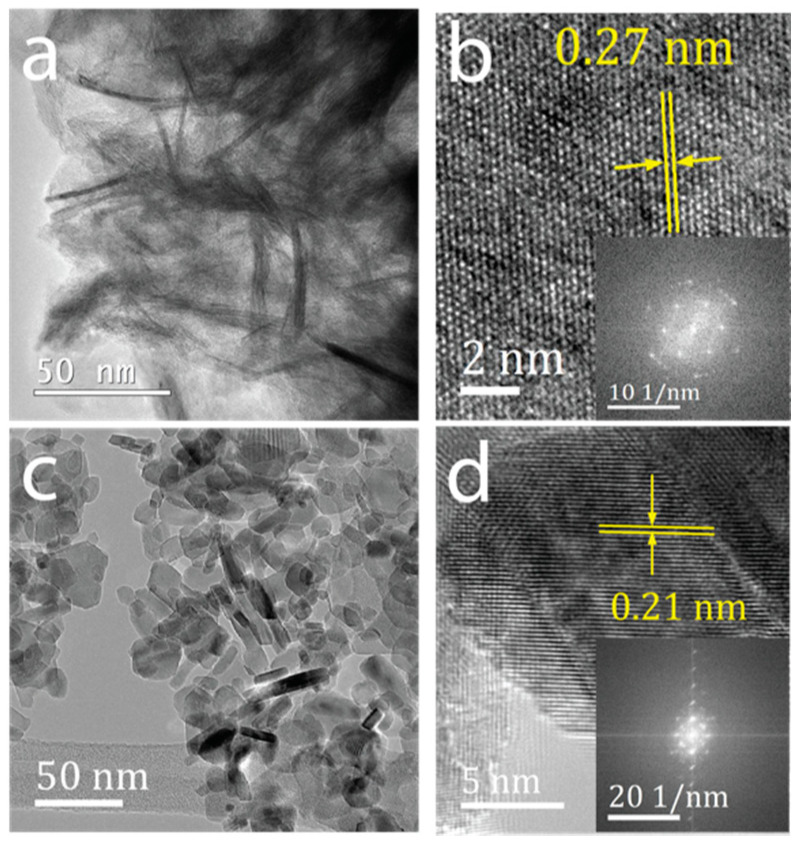
(**a**) Low and (**b**) high resolution TEM image of *β*-Ni(OH)_2_; (**c**) low and (**d**) high resolution TEM image of NiO. The insets in b and d show the fast Fourier transformations obtained from the corresponding images.

A facile solution-based approach was used for the synthesis of *β*-Ni(OH)_2_ and NiO nanoparticles using triethylamine as the hydrolysing agent of the aqueous solution of Ni^2+^ ions. Figure 1 shows the morphological characteristics of both particles. TEM image of the *β*-Ni(OH)_2_ showed thin 2-dimensional (2D) sheet-like morphology (Figure 1a). Following the process of calcination to convert *β*-Ni(OH)_2_ to NiO, the thin crumbled sheet-like morphology changed to irregular morphologies. However, the resulting NiO still retained the overall 2D sheet-like structures (Figure 1c). This morphological change may be attributed to the sintering effect during the calcination of the hydroxide phase to the corresponding oxide. The high crystallinity of both *β*-Ni(OH)_2_ (Figure 1b) and NiO (Figure 1d) is evident from lattice fringes observed under HR-TEM. The images show that in the case of *β*-Ni(OH)_2_, the interlayer spacing between the neighbouring fringes was ca. 0.27 nm which corresponds to the (010) crystal plane of the hexagonal *β*-Ni(OH)_2_ [34]. For NiO, the observed lattice spacing was ca. 0.21 nm corresponding to the (200) crystal plane of the cubic NiO [35]. Fast Fourier transformations (FFT) of the images reveal a well-defined diffraction pattern corresponding to the hexagonal and cubic phase of the *β*-Ni(OH)_2_ (inset in Figure 1b) and NiO, respectively (inset in Figure 1d), attesting to the high crystallinity of these materials. The distribution of the different chemical species in these particles was further assessed, where the EDX spectra (Figure S1) obtained from both nanomaterials showed characteristic energy lines corresponding to Ni and O, while the elemental maps (Figures S2 and S3) showed an even distribution of both elements across the nanomaterial surface. 

**Figure 2:**
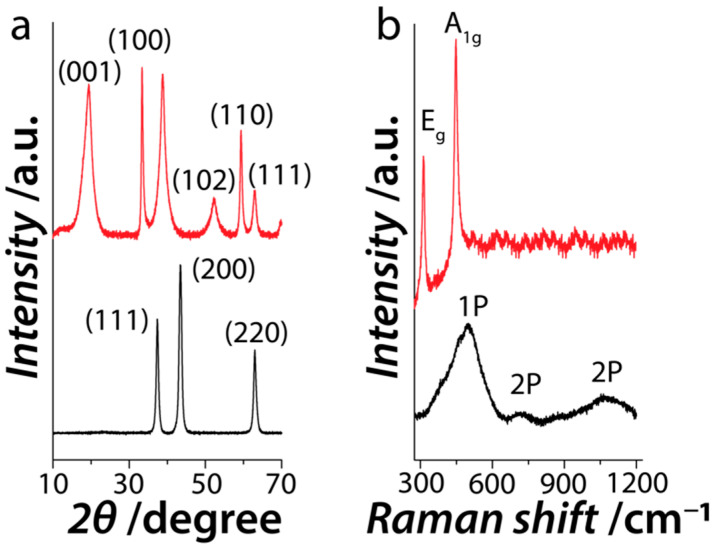
(**a**) XRD patterns and (**b**) Raman spectra obtained from the hexagonal *β*-Ni(OH)_2_ (red line) and cubic NiO (black line).

XRD analysis (Figure 2a) confirmed that the greenish powder was indeed the hexagonal *β*-Ni(OH)_2_ phase (JCPDS: 74-2075) and the intense peak of the (100) suggests that the *β*-Ni(OH)_2_ nanosheets preferentially grow along the (100) plane, confirming their 2D morphology [35,36,37,38]. XRD pattern obtained from the black powder confirmed the face-centered cubic phase of NiO (JCPDS no 78-0423) with the most intense (200) plane supporting its 2D morphology, while the absence of any additional peak supporting a phase pure material. The Raman spectrum of *β*-Ni(OH)_2_ showed peaks at 312.6 and 448.3 cm^−1^ which are the characteristic lattice vibrational *Eg* and *A_1g_* modes of the β-phase of nickel hydroxide (Figure 2b) [39,40]. Similarly, NiO showed three characteristic broad vibrational bands corresponding to the one-phonon (1P) mode at 497.4 cm^−1^ as well as the two-phonon (2P) modes at 726 cm^−1^ (transverse optical) and 1068 cm^−1^ (longitudinal optical). The broadening of the Raman bands in the case of NiO suggests the presence of structural defects or surface effects, both of which are well documented in the literature [40,41]. In such cases, the 1P band becomes pronounced with concomitant disappearance of the 906 cm^−1^ band [41]. 

**Figure 3:**
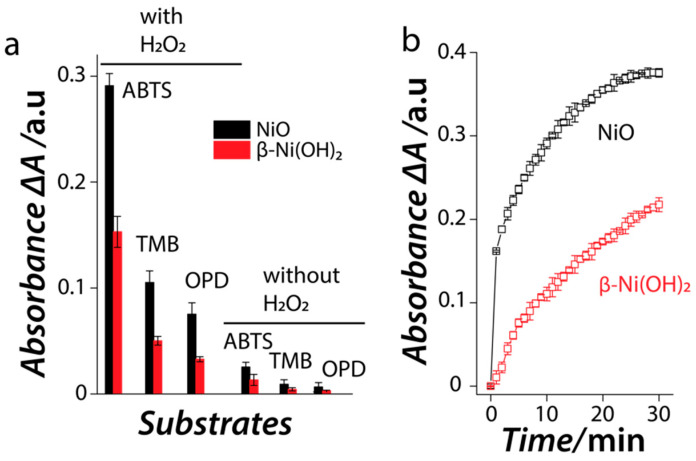
(**a**) The ability of the Ni-based nanozymes to promote the oxidation of different substrates (0.5 mM) in the presence and absence of H_2_O_2_ (10 mM); (**b**) the peroxidase-mimic catalytic activity of NiO and *β*-Ni(OH)_2_ nanozymes as a function of time at pH 3 and 30 °C.

To establish the enzyme-mimic catalytic activity of the *β*-Ni(OH)_2_ and NiO nanomaterials, we first assessed their ability to oxidize chromogenic peroxidase substrates ABTS, TMB and OPD in the presence and absence of H_2_O_2_. In the case of oxidase-mimic activity, the nanomaterials would be able to catalyze the oxidation of these substrates in the absence of H_2_O_2_. However, the need for additional H_2_O_2_ to drive the oxidation of the chromogenic substrates would suggest that the nanomaterials mimic the natural peroxidase enzyme. Figure 3a shows that both nanomaterials could catalyze the oxidation of peroxidase substrate (ABTS) in the absence of H_2_O_2_, however, this oxidase-mimic activity is quite weak. A significant increase in the substrate oxidation (more than an order of magnitude increase) in the presence of H_2_O_2_ suggests that these materials predominantly mimic the peroxidase enzyme. It was also observed that both nanomaterials showed the highest catalytic activity towards ABTS oxidation, while they could oxidize TMB and OPD with lower efficiencies. This is interesting as nanozymes have been reported to have some degree of specificity towards substrates. This specificity is typically attributed to the surface charge on the nanozyme where positively charged nanomaterials are shown to have a high affinity towards negatively charged ABTS substrate while negatively charged nanomaterials show higher affinity for positively charged TMB and OPD substrates [42,43,44,45]. In the current study, zeta potential measurements revealed that both the *β*-Ni(OH)_2_ and NiO nanomaterials have a zeta potential value of +11.4 mV and +21.4 mV, respectively at the physiological pH. Given that the catalytic activity of these nanomaterials was assessed at pH 3, the zeta potential values at this pH were +27.6 mV and +41.1 mV, respectively. This observation corroborates well with our previous work, where we used triethylamine for the synthesis of Gd(OH)_3_ and Gd_2_O_3_ [21]. Based on the high positive charge on the surface, it is unsurprising that these materials show high catalytic activity for oxidizing ABTS substrate (pKa ~1.5). This supports the ongoing hypothesis that the substrate affinity in nanozymes most likely results from electrostatic interactions between the nanozyme and the substrate. Another observation is that both nanozymes also show the ability to oxidize positively charged TMB and OPD substrates, but with lower efficiency than ABTS. This is interesting as the high positive charge on the surface of the nanozyme and protonated form of TMB and OPD substrates in acidic medium should not lead to an electrostatic attraction (pK_a_ of TMB and OPD are ~4.2 and ~4.4, respectively). This suggests that the surface charge of the nanozyme is not the only factor that governs the ability of a nanozyme to oxidize different substrates. One such factor was recently pointed out where the catalytic activity of nanozymes was suggested to be dependent on their ability to cleave the leaving group in the substrates [29]. This suggests that there is a complex interplay between the substrate leaving group, the surface charge, and the inherent property of the nanomaterial in dictating the ability of a nanozyme to oxidize different substrates. Further, between the two materials, the NiO nanozyme showed higher catalytic activity than *β*-Ni(OH)_2_. Considering that both nanozymes showed high specificity in catalysing the oxidation of ABTS substrate, further studies were performed using ABTS as the substrate of choice.

A time-dependent kinetic study of the catalytic reaction between the nanozymes and ABTS/H_2_O_2_ substrates showed that in both cases the activity proceeds rapidly at initial time points (Figure 3b). In the case of NiO nanozyme, the activity saturates within ~30 min of the reaction, while the *β*-Ni(OH)_2_ nanozyme showed a slow but steady increase in the catalytic activity as a function of time. To ensure that the catalytic activity was an inherent property of the two nanozymes and that there was no role of Ni ions potentially leached from the nanoparticles, control experiments were performed where the leached ion solution obtained after incubating the nanozymes under similar conditions (10 mM glycine HCl buffer (pH 3) at 30 °C for 30 min) was used as a catalyst, which showed minimal catalytic activity (Figure S4a). Further, we also used five known concentrations of Ni^2+^ ions as a catalyst under similar conditions which showed no catalytic activity (Figure S4b). This suggests a direct contribution of the nanozymes in promoting the catalytic reaction. Additionally, the concentration of the leached Ni ions was also determined using AES. Minimal leaching was observed in all cases, which ascertained the high stability of these materials during catalysis (Figure S5). 

Important reaction parameters such as the nanozyme concentration, pH, and temperature were then optimized as these parameters have the potential to modulate the catalytic efficiency of a catalyst, a property akin to natural enzymes and other nanozymes [46,47]. The catalytic activity of both nanozymes increased with the increasing nanozyme concentration (Figure S6a). The NiO nanozyme showed maximum catalytic activity at 1.5 mM Ni equivalent concentration where further increase in the Ni concentration had minimal effect on the catalytic efficiency. In contrast, the catalytic activity of the *β*-Ni(OH)_2_ nanozyme continued to increase up to the highest tested concentration (1.75 mM Ni). Both nanozymes showed optimal activity at 30 °C (Figure S6b) and pH 3 (Figure S6c). Further increase in the temperature or moving from acidic conditions towards neutral or alkaline pH resulted in the loss of catalytic activity. 

Further studies were performed using the optimal conditions for both nanozymes—Ni concentration of 1.5 mM, pH 3 and temperature of 30 °C. Using the enzyme kinetic theory, we also calculated the Michaelis constant (*K_m_*) and maximum initial velocity (*V_max_*) for both nanozymes. For this, the catalytic reaction was monitored by varying one substrate concentration at a time while keeping the concentration of the second substrate fixed. A plot of the initial velocity (*V_0_*) vs. the substrate concentration showed typical Michaelis-Menten curves (Figure S7). These values were then used for the Lineweaver-Burk plot to determine the *K_m_* and *V_max_* (Table 1). The calculated *K_m_* values suggest that the NiO nanozyme has a higher affinity for H_2_O_2_ substrate than ABTS, while the *β*-Ni(OH)_2_ nanozyme has a higher affinity for ABTS substrate. This is an interesting observation, as most of the inorganic nanozymes show a high affinity for the chromogenic substrate, while the lower affinity to H_2_O_2_ [21,24,48,49,50]. In the current study, NiO shows a higher affinity for H_2_O_2_ suggesting that it is the affinity of the nanozyme towards the chromogenic substrate that limits the catalytic reaction. The comparison of the *V_max_* values of two nanozymes towards ABTS oxidation suggests that the NiO nanozyme is a better catalyst than *β*-Ni(OH)_2_ nanozyme. Considering that NiO nanozyme is a superior peroxidase-mimic, further interaction studies were performed using NiO as a model nanozyme to understand its interaction with L-cysteine amino acid. 

**Figure 4:**
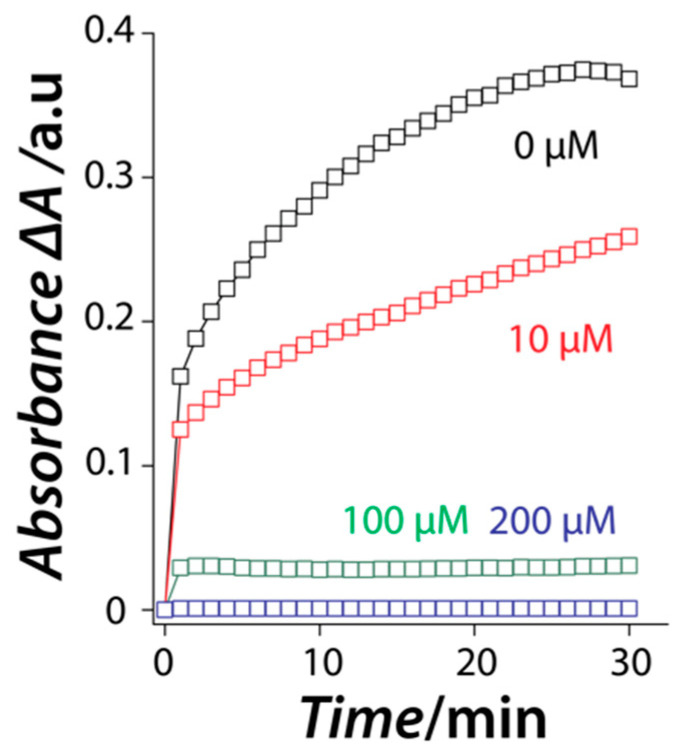
The inhibition of the peroxidase-mimic catalytic activity of the NiO nanozyme as a function of time in the absence and presence of different L-cysteine concentrations (Reaction conditions: ABTS—0.5 mM; H_2_O_2_—10 mM; pH—3.0, temperature—30 °C).

Our previous work with Gd-based nanozymes showed that L-cysteine amino acid acts as a reversible inhibitor [51,52]. In that case, L-cysteine did not act as a catalyst poison but instead, it interacted with the nanozyme through a competitive inhibition process where there was a competition between L-cysteine and the peroxidase substrates to bind to the surface of the nanozyme. The first line of thought in the current case of Ni-based nanozymes was—would a similar reversible interaction occur when NiO nanozyme is exposed to L-cysteine? For this, three independent concentrations of L-cysteine were exposed to a fixed concentration of NiO nanozyme in the presence of ABTS/H_2_O_2_ and the reaction was monitored as a function of time. To our surprise, at all the tested L-cysteine concentrations, the nanozyme could initially catalyze the oxidation of ABTS substrate with an almost equivalent pace irrespective of the concentration of L-cysteine (Figure 4). However, after this initial burst phase, the reaction saturates with increasing rapidity with an increase in L-cysteine concentration. This observation is in stark contrast to our previous study on Gd-based nanozymes where the activity was completely blocked initially in the presence of L-cysteine and subsequently regained with time even at extremely high L-cysteine concentrations (Figure S8) [21]. Interestingly, in the current case, the catalytic activity of the NiO nanozyme does not improve with time in cases where L-cysteine concentration is high enough to block the initial activity (100 and 200 µM). This suggests that L-cysteine may act as an irreversible inhibitor of the catalytic activity of the NiO nanozyme. 

This would mean that a stable nanozyme-cysteine complex is formed as soon as the NiO nanozyme is exposed to L-cysteine. The formation of this stable complex will significantly deplete the population of free L-cysteine (inhibitor) in the reaction. Based on enzyme kinetic theory, such inhibitors bind to the natural enzyme with an apparent affinity close to the concentration of the active sites of the enzyme, i.e., one inhibitor molecule inactivating a single enzyme molecule. This implies that as the concentration of the inhibitor is increased, at a particular inhibitor concentration, one would observe a complete loss of the enzyme activity. In nanozymes, the surface atoms are considered to play the role of the active site, which is likely to result in multiple active sites within a single nanozyme particle. This means that a nanozyme-driven reaction would require very high concentrations of the inhibitor molecule to completely inactivate a single nanozyme. If we consider the potential impact of this scenario on the steady-state kinetics (*K_m_* and *V_max_*) of the nanozyme, we should expect to see no change in the *K_m_*, as the irreversible binding of L-cysteine to the nanozyme in the initial phase should not influence the simultaneous binding of substrate molecules to the surface of the nanozyme. However, given that fewer free surface atoms are consequently available to interact with the substrate, we should see a decrease in the *V_max_* for nanozyme-mediated substrate oxidation. To validate this hypothesis, we calculated the *K_m_* and *V_max_* in the presence and absence of L-cysteine (Figure S9). In line with our hypothesis, we do indeed observe a decrease in the *V_max_* for the substrates ABTS and H_2_O_2_ as we increase the concentration of L-cysteine, while the *K_m_* remains constant (Figure S9 and Table S1).

To further validate the irreversible inhibition of NiO nanozyme by L-cysteine, we employed FTIR spectroscopy (Figure S10). The above hypothesis relies on the premise that L-cysteine permanently binds to the surface of the nanozyme, which should become evident from FTIR studies. The FTIR of the NiO showed a broad peak at ca. 3540 cm^−1^ associated with the asymmetric and symmetric stretching vibrations of the −OH group (absorbed water molecules and the surface hydroxyls) [53,54,55]. The FTIR spectrum of pristine L-cysteine amino acid showed characteristic peaks of the −COO stretch (1581 cm^−1^, 1422 cm^−1^), -SH bend and stretching (2550 cm^−1^ and 943 cm^−1^) as well as those from the amine functional group (3184 cm^−1^) [56,57]. Following the interaction of the L-cysteine and NiO nanozyme, the particles were extracted and washed. The FTIR spectrum of the washed nanozyme showed additional strong peaks arising from the -SH bend and -COO stretch, which were absent in the case of pristine NiO nanozyme [56,57]. These observations affirm the strong binding ability of L-cysteine to the NiO nanozyme, supporting the formation of a stable nanozyme-inhibitor complex. 

Classical enzyme kinetic models suggest that irreversible inhibitors or tight binding inhibitors can display a non-competitive phenotype [57]. In the current case, based on steady state kinetics, the inhibitory activity of L-cysteine shows features corresponding to non-competitive inhibition, while the time-dependent activity shows that the catalytic activity of the nanozyme does not recover with time. Both of these aspects together confirms that L-cysteine in fact acts as an irreversible inhibitor of the NiO nanozyme activity. To further understand if the interaction of L-cysteine with NiO leads to irreversible changes in the nanozyme, we also performed XRD studies following the exposure to L-cysteine of NiO. As evident from the XRD spectra before and after L-cysteine exposure (Figure S11), we observe no change in the chemical nature of the NiO nanozyme. 

**Figure 5:**
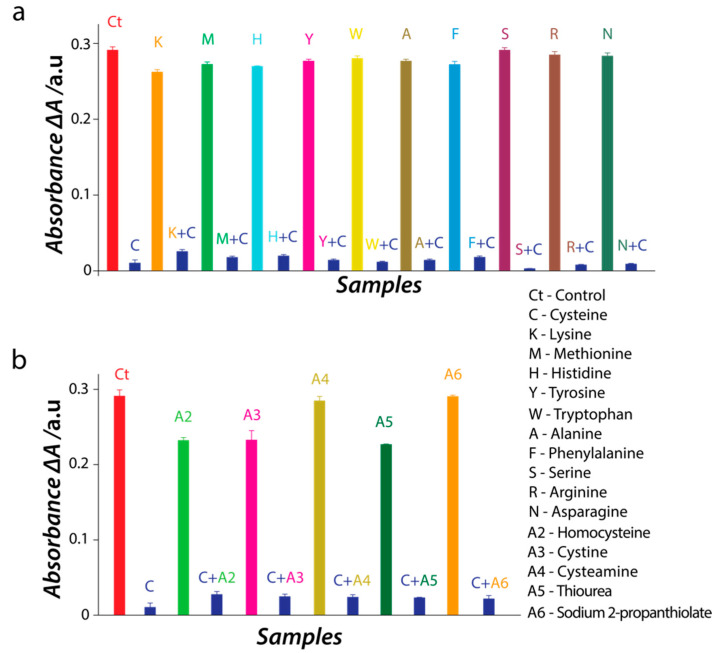
(**a**) The inhibition of the peroxidase-mimic catalytic activity of NiO nanozyme in the presence of different amino acids either independently or in combination with L-cysteine (200 μM concentration of amino acids and L-cysteine); (**b**) the inhibition of peroxidase-mimic catalytic activity of NiO nanozyme in the presence of different sulfur containing compounds (200 μM) either independently or in combination with L-cysteine.

Having established that L-cysteine binds irreversibly to the NiO nanozyme, it was pertinent to understand if L-cysteine is the only amino acid that has this capability. To understand this aspect, we exposed the NiO nanozyme to a fixed concentration of other commonly occurring amino acids including L-methionine which is another sulfur-containing amino acid. In all cases, we observed minimal interaction of the amino acid with the nanozyme resulting in no or minimal loss of the catalytic activity (Figure 5a). In contrast, if the same amino acids were mixed with a fixed concentration of L-cysteine, we observed an immediate loss of the catalytic activity. Further studies were performed by exposing the NiO nanozyme to several thiolic compounds (cystine, cystamine, thiourea, sodium 2-propanthiolate, and homocysteine). We observed that these sulfur containing molecules resulted in some loss of the nanozyme activity (Figure 5b). However, the influence of these thiolic compounds on the activity of NiO nanozyme is remarkably smaller in contrast to the near-complete loss of activity induced by L-cysteine. 

Importantly, in the above experiments, the NiO nanozyme was first exposed to L-cysteine or other interfering molecules prior to the addition of the chromogenic substrate ABTS and the co-substrate H_2_O_2_. It is, however, known that H_2_O_2_ can oxidize L-cysteine to form cysteine [58]. This catalytic activity is significantly faster at neutral or alkaline pH. In the current case, if this were to occur, then we should not have observed any inhibition of the nanozyme activity, especially considering that cystine itself didn’t significantly block the nanozyme activity. To validate the influence of H_2_O_2_ on L-cysteine in the presence of the nanozyme, we performed additional experiments in which, we first exposed L-cysteine to H_2_O_2_ for 10 min and then added the NiO nanozyme and ABTS. The plot of ABTS oxidation as a function of time shows that the oxidation of ABTS is only marginally inhibited in this case (Figure S12). This suggests that the formation of a stable cysteine-nanozyme complex protects L-cysteine against H_2_O_2_-induced oxidation. This experiment also validates that it is irreversible inhibition of the nanozyme activity by L-cysteine, and not a potential parallel reaction pathway involving H_2_O_2_ consumption during L-cysteine oxidation that results in the reduction of the nanozyme activity.

**Figure 6:**
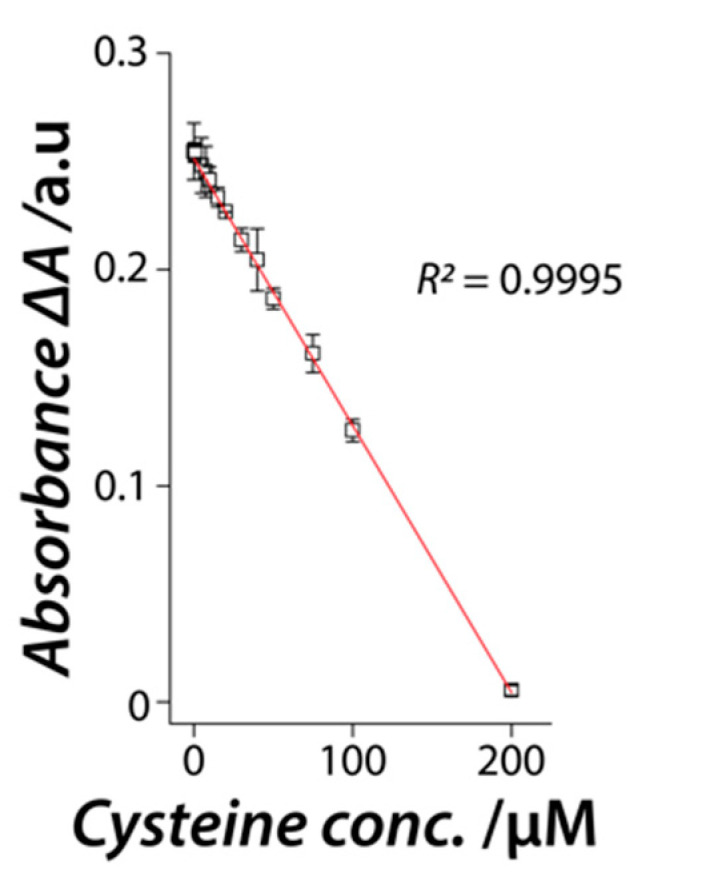
A plot of the change in absorbance due to the oxidation of ABTS substrate as a result of the catalytic activity of NiO nanozyme vs. L-cysteine concentration showing a linear regression correlation.

We, then employed this high specificity of the NiO nanozyme to interact with L-cysteine for the quantitative detection of L-cysteine. Different concentrations of L-cysteine were exposed to a fixed concentration of the NiO nanozyme along with both ABTS and H_2_O_2_ substrates. The change in absorbance due to the peroxidase-mimic catalytic activity (with respect to activity without L-cysteine) was plotted against the L-cysteine concentration (Figure 6). 

A linear correlation was observed between 0.1 μM–200 μM L-cysteine concentration, beyond which, the activity saturated. Ten independent experiments with three different concentrations (lowest, mid and highest) of L-cysteine in the dynamic linear range were used to determine the accuracy and precision of L-cysteine detection. The results showed over 96.5% precision for all concentrations (0.1 μM—96.96%, 50 μM—96.67%, and 200 μM—98.45%) and 100% accuracy at a significance of 10% confidence level. This outlines the robustness of the NiO nanozyme system as a potential L-cysteine sensor.

## 4. Conclusions

The current study establishes the peroxidase-mimic catalytic activity of two Ni-based 2D nanomaterials viz. NiO and *β*-Ni(OH)_2_. Both nanozymes showed high specificity towards oxidation of negatively charged ABTS substrate over other positively charged substrates. The activity of the oxide phase was found superior to the hydroxide phase. Importantly, our study for the first-time establishes L-cysteine as an irreversible inhibitor to permanently block the catalytic activity of the NiO nanozyme. Given that surface atoms play the role of active sites in nanozymes, the inhibition of activity was found to be dependent on the concentration of L-cysteine. In depth analysis using enzyme kinetic theory confirmed that the addition of L-cysteine to the reaction did not affect the affinity of the nanozyme to its substrates but only reduced the catalytic efficiency. This ability to block the enzyme-mimic activity was specific to L-cysteine amino acid. The specific nature of this interaction allowed the fabrication of a simple colorimetric sensor for L-cysteine using the NiO nanozyme system. This sensor allowed us to detect L-cysteine in the presence of other amino acids as contaminants either independently or in a mixture. This work opens a new avenue of research to find molecules that bind either reversibly or irreversibly to nanozymes bringing them one-step closer to natural enzymes.

## Figures and Tables

**Table 1:** Apparent enzyme kinetic parameters calculated for the NiO and *β*-Ni(OH)_2_ nanozymes at pH 3, 30 °C and Ni ion concentration of 1.5 mM.

	NiO		*β*-Ni(OH)_2_	
	ABTS	H_2_O_2_	ABTS	H_2_O_2_
*K_m_* (mM)	17.5	14.8	7	14
*V_max_* (mM/s)	5.3 × 10^−4^	1.1 × 10^−4^	2.3 × 10^−4^	8.7 × 10^−4^

## Data Availability

No new data were created or analyzed in this study. Data sharing is not applicable to this article.

## Supplementary Materials

The following are available online at https://www.mdpi.com/article/10.3390/nano11051285/s1, Figure S1. The EDX spectrum obtained from (**a**) *β*-Ni(OH)_2_ and (**b**) NiO, Figure S2. EDS elemental maps obtained from a single *β*-Ni(OH)_2_ particle, Figure S3. EDS elemental maps obtained from a cluster of NiO particles, Figure S4. (**a**) A plot of the absorbance vs. the reaction time of the two nanozymes and leached ions; (**b**) a plot of the absorbance vs. the reaction time of the two nanozymes and known concentrations of Ni^2+^ ions as a catalyst, Figure S5. The concentration of Ni ion in solution after the incubation of the Ni(OH)_2_ (red bars) and NiO nanozyme (black bars) in pH 3 buffer for 30 min, followed by centrifugation to obtain nanozyme as the pellet and potentially leached Ni^2+^ ions in the supernatant, Figure S6. The effect of the (**a**) nanozyme concentration, (**b**) temperature, and (**c**) pH on the peroxidase-mimic catalytic activity of the Ni(OH)_2_ (red bars) and NiO (black bars) nanozyme. The error bars represent the standard deviation obtained from three independent experiments, Figure S7. (**a**,**b**) Michaelis-Menten and (**c**,**d**) Lineweaver-Burk plots for the *β*-Ni(OH)_2_ nanozyme (red) and NiO nanozyme (black). The error bars represent the standard deviations obtained from three independent experiments. The non-linear fitting for Michaelis-Menten and linear fitting for Lineweaver-Burk was performed using OriginPro 2016. (Reaction conditions include pH 3, 30 °C and Ni concentration of 1.5 mM; when ABTS concentration was changed, H_2_O_2_ concentration was maintained at 10 mM; when H_2_O_2_ concentration was changed, ABTS concentration was maintained at 0.5 mM), Figure S8. Change in the nanozyme activity of Gd(OH)_3_ nanorods with increasing concentration of L-Cys as a function of time, Figure S9. Lineweaver-Burk (LB) plots obtained for the NiO nanozyme in the presence of different concentrations of L-cysteine inhibitor. (**a**) shows the LB plots with varying ABTS concentration at a fixed 10 mM H_2_O_2_ concentration while (**b**) shows the LB plots with varying H_2_O_2_ concentration at a fixed 0.5 mM ABTS concentration. The error bars represent the standard deviations obtained from three independent experiments. The linear fitting was performed using OriginPro 2016, Figure S10. FTIR spectral analysis for NiO nanozyme (black), L-cysteine (red) and NiO and L-cysteine interaction (blue), Figure S11. XRD spectra obtained from NiO nanozyme before and after its exposure to L-cysteine, Figure S12. A plot of the change in absorbance as a function of time for (i) pristine NiO nanozyme exposed to ABTS and H_2_O_2_; (ii) L-cysteine exposed to H_2_O_2_ for 10 min followed by the addition of ABTS and NiO and NiO exposed to L-cysteine for 10 min followed by the addition of ABTS and H_2_O_2_, Table S1. Comparison of apparent enzyme kinetic parameters for the NiO nanozyme in absence and presence of different concentrations of L-cysteine.
