# Supplementary material for: L-Cysteine as an Irreversible Inhibitor of the Peroxidase-Mimic Catalytic Activity of 2-Dimensional Ni-Based Nanozymes
Source: Nanomaterials (Basel). 2021 May 13;11(5):1285. doi: 10.3390/nano11051285 (PMC8153149; doi:10.3390/nano11051285)
Supplement: Supplementary file 1 [file nanomaterials-11-01285-s001.zip › nanomaterials-1148775-supplementary.pdf]

nanomaterials

MDPI

Supplementary Materials

L-Cysteine as an Irreversible Inhibitor of the Peroxidase-Mimic Catalytic Activity of 2-Dimensional Ni-Based Nanozymes

Piyumi Dinusha Liyanage †, Pabudi Weerathunge †, Mandeep Singh †, Vipul Bansal * and Rajesh Ramanathan *

Ian Potter NanoBioSensing Facility, NanoBiotechnology Research Laboratory (NBRL), School of Science, RMIT University, GPO Box 2476, Melbourne VIC 3000, Australia; s3437386@student.rmit.edu.au (P.D.L.); pabudi.weerathunge@rmit.edu.au (P.W.); mandeep.singh@rmit.edu.au (M.S.)

* Correspondence: vipul.bansal@rmit.edu.au (V.B.); rajesh.ramanathan@rmit.edu.au (R.R.); Tel.: +61-3-9925-2121 (V.B.); +61-3-9925-2887 (R.R.)

† Equal contributing authors.

Characterization of $Ni(OH)_2$ and NiO.

Both nanomaterials were thoroughly characterized using a suite of materials characterization tools including transmission electron microscopy (TEM) and high resolution TEM (HR-TEM) using JEOL 1010 and JEOL 2100F (FEG system) TEM instruments, operated at an accelerating voltage of 100 kV and 80 kV, respectively. The nanomaterials were drop casted on a holey carbon grid for TEM studies. energy dispersive X-ray (EDX) spectra and corresponding chemical maps were acquired on the 2100F TEM instrument in STEM mode using an Oxford X-max^n 80T detector. X-ray diffraction (XRD) patterns was obtained using Bruker AXS D4 Endeavour – wide angle XRD with Cu Kα radiation (λ: 1.5406 Å). X-ray photoemission spectroscopy (XPS) measurements were carried out using Thermo K-Alpha XPS instrument operating at a pressure better than 1×10^{-8} Torr (1 Torr=1.333×10^{2} Pa). The core level spectra of different elements were recorded with unmonochromatized Al Kα radiation (photon energy of 1486.7 eV) at pass energy of 20 eV and an electron take off angle of 90° with an overall resolution of 0.1 eV. The core level spectra were background corrected using the Shirley algorithm and chemically distinct species were resolved using nonlinear least squares fitting procedure. The binding energies (BEs) were aligned with the adventitious C 1*s* binding energy of 285 eV. The quantification of Ni ions present in both nanomaterials was carried out using Agilent 4200 microwave plasma – atomic emission spectrometer (MP-AES) after digesting the nanomaterials in concentrated nitric acid. The absorbance spectra were obtained using Envision multimode plate reader (Perkin Elmer). Raman spectra were obtained using a Horiba LabRAM HR Evolution micro-Raman system equipped with 532 nm laser (0.5 μm lateral resolution, 0.25 s exposure) and a 100× objective. Raman spectra was background corrected using an in-house developed smoothing free algorithm. Surface charge on the nanoparticles was measured using a Malvern Nano-Zs/Zen3600 zetasizer instrument.

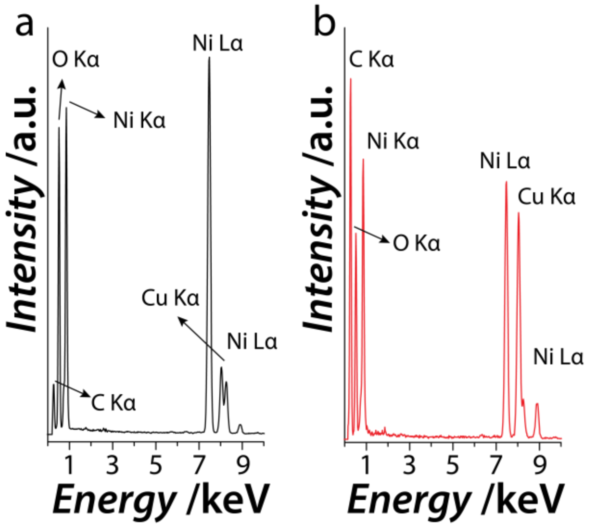

Figure S1. The EDX spectrum obtained from (**a**) β-$Ni(OH)_2$ and (**b**) NiO.

Following the synthesis of both Ni-based nanomaterials, TEM images reveal that the β-$Ni(OH)_2$ particles show thin plate-like morphology while the NiO shows irregular thin sheet-like morphology (Figure 1). The EDX spectra obtained from both the β-$Ni(OH)_2$ (Figure S1a) and NiO (Figure S1b) show the characteristic energy lines corresponding to Ni and O elements. We also observe energy lines corresponding to C and Cu from the underlying carbon-coated copper grid. The EDS elemental map of Ni and O as well as a stacked image obtained from the β-$Ni(OH)_2$ shows even distribution of the elements across the surface of the material (Figure S2). Similar distribution was observed for the NiO particle (Figure S3).

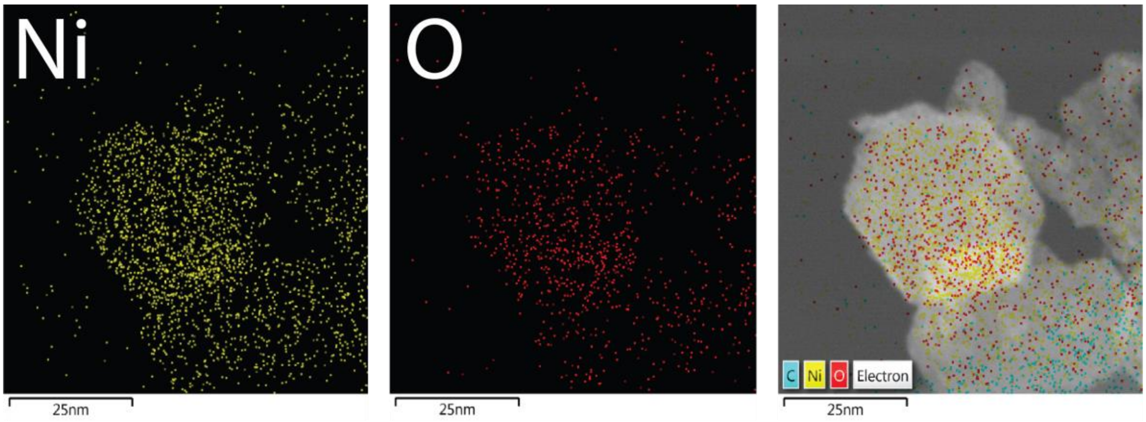

Figure S2. EDS elemental maps obtained from a single β-$Ni(OH)_2$ particle.

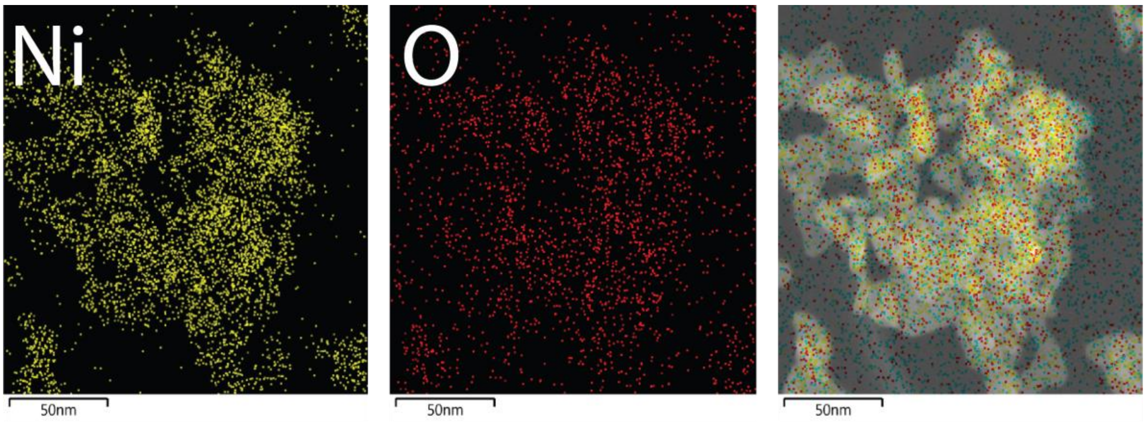

Figure S3. EDS elemental maps obtained from a cluster of NiO particles.

Evaluation of the enzyme-mimic catalytic activity of the *β*-$Ni(OH)_2$ and NiO nanozymes.

The enzyme-mimicking catalytic activity of both nanozymes was first evaluated by assessing their ability to oxidize ABTS, TMB and OPD in the presence and absence of H_2O_2. As shown in Figure 3a, both nanozymes showed the ability to promote the oxidation of the ABTS, TMB and OPD specifically in the presence of H_2O_2, while minimal activity was observed in the absence of H_2O_2 substrate. This suggests that both materials mimic the activity of natural peroxidase enzyme and not oxidase enzyme. While both the Ni-based nanozymes showed high propensity to oxidize ABTS, it was important to ensure that the catalytic activity was indeed an inherent property of the nanomaterial and not achieved due to leached metal ions. For this, each nanomaterial was first incubated in 10 mM glycine HCl buffer (pH 3) at 30 °C for 30 min, independently. The nanomaterial was removed by high speed centrifugation and the resulting supernatant containing the leached ion was used as a catalyst. Figure S4 shows minimal catalytic activity from the leached ions suggesting a direct contribution of the nanozyme in promoting the catalytic reaction.

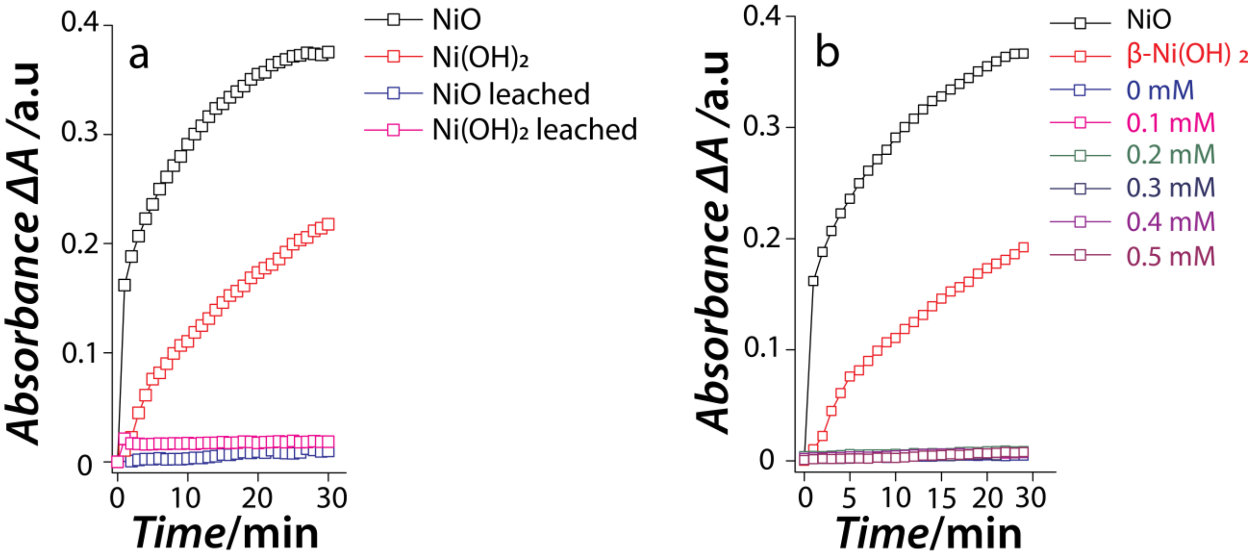

Figure S4. (**a**) A plot of the absorbance vs. the reaction time of the two nanozymes and leached ions; (**b**) a plot of the absorbance vs. the reaction time of the two nanozymes and known concentrations of Ni^{2+} ions as a catalyst.

While it was important to establish the inherent role of the nanozyme in promoting the catalytic reaction, it was also important to understand the stability of the nanozyme in these conditions (incubation of the nanozyme in glycine HCl buffer - pH 3). The Ni ion content in the supernatant and pellet obtained after the incubation of each nanozyme was estimated using AES. Figure S5 shows that the concentration of Ni in the pellet was consistent with the original starting concentration, while the Ni ion concentration in the supernatant was insignificant. This outlines the high stability of both nanozymes during the catalytic reaction conditions.

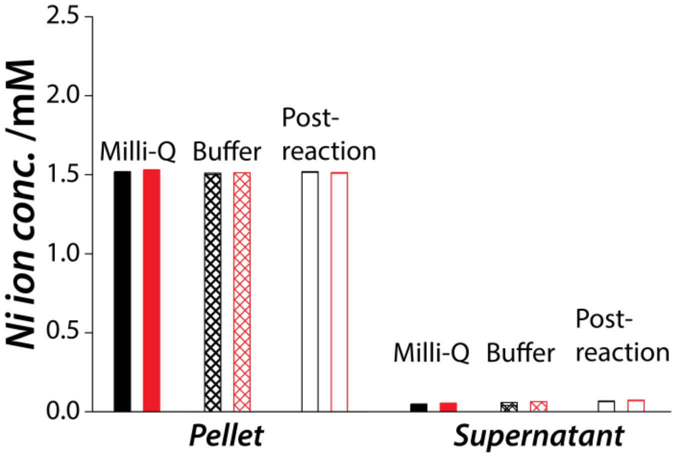

Figure S5. The concentration of Ni ion in solution after the incubation of the $Ni(OH)_2$ (red bars) and NiO nanozyme (black bars) in pH 3 buffer for 30 min, followed by centrifugation to obtain nanozyme as the pellet and potentially leached Ni^{2+} ions in the supernatant.

The catalytic activity of the nanozymes is dependent on the reaction conditions, a property akin to natural enzymes as well as other nanozymes.[1, 2] The reaction conditions including nanoparticle concentration, pH, and temperature were optimized. As shown in Figure S6, the catalytic activity of both nanozymes increases as a function of nanozyme (equivalent Ni ions) concentration (Figure S6a). For the β-$Ni(OH)_2$ nanozyme, the catalytic activity still continues to increase up to 1.75 mM Ni, the highest tested concentration. In contrast, the catalytic activity of the NiO nanozyme saturates at ca. 1.5 mM Ni concentration. A temperature-dependent activity profile shows that both nanozymes have optimal catalytic activity at 30 °C (Figure S6b) while the activity was highest at pH 3 (Figure S6c).

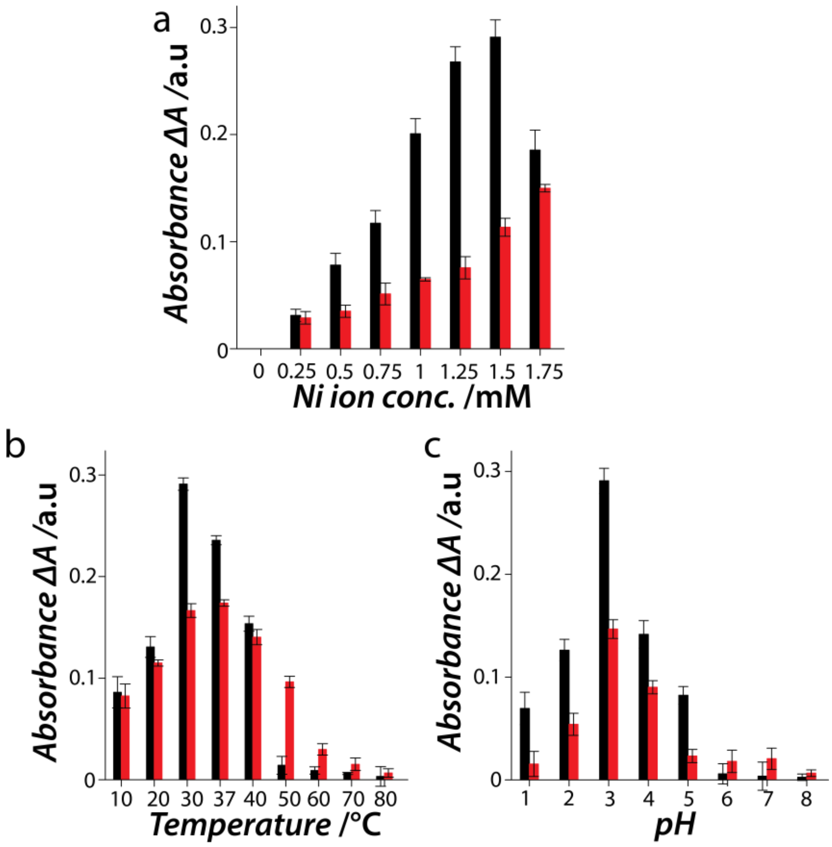

Figure S6. The effect of the (**a**) nanozyme concentration, (**b**) temperature, and (**c**) pH on the peroxidase-mimic catalytic activity of the $Ni(OH)_2$ (red bars) and NiO (black bars) nanozyme. The error bars represent the standard deviation obtained from three independent experiments.

The enzyme kinetic parameters including the determination of the Michaelis-Menten constant (K_m) and maximum initial velocity (V_{max}) outlines the affinity and catalytic efficiency of the nanozyme, respectively.[3] For this, the catalytic reaction was monitored by varying one substrate concentration at a time while keeping concentration of second substrate fixed at a fixed concentration of the nanozyme. A plot of the initial velocity (V_0) *vs*. the substrate concentration showed typical Michaelis-Menten curves (Figure S7a–b), a feature akin to natural enzymes. An algebraically transformed variation of the Michaelis–Menten equation – commonly referred to as Lineweaver-Burk plot further allowed us to determine the K_m and V_{max} for both nanozymes (Figure S7c–d). Table S1 shows the K_m for both ABTS and H_2O_2 substrates, where NiO shows higher affinity for H_2O_2 while β-$Ni(OH)_2$ shows higher affinity for ABTS substrate. The V_{max} values suggest that the NiO nanozyme is a better catalyst than β-$Ni(OH)_2$ nanozyme. Therefore, further interaction studies were performed using NiO as a model nanozyme catalyst.

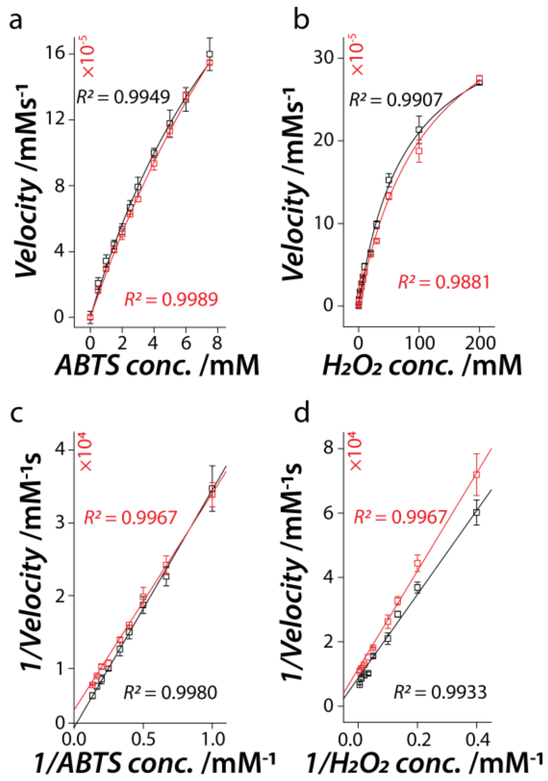

Figure S7. (**a**, **b**) Michaelis-Menten and (**c**, **d**) Lineweaver-Burk plots for the β-$Ni(OH)_2$ nanozyme (red) and NiO nanozyme (black). The error bars represent the standard deviations obtained from three independent experiments. The non-linear fitting for Michaelis-Menten and linear fitting for Lineweaver-Burk was performed using OriginPro 2016. (Reaction conditions include pH 3, 30 °C and Ni concentration of 1.5 mM; when ABTS concentration was changed, H_2O_2 concentration was maintained at 10 mM; when H_2O_2 concentration was changed, ABTS concentration was maintained at 0.5 mM).

The ability of certain molecules to either permanently or temporarily inhibit the catalytic activity of natural enzymes are known as enzyme inhibition and these molecules are referred as inhibitors. It has been shown that certain amino acids have the capability to temporarily inhibit the catalytic activity of nanozymes such as that in the case of Gd-based nanozymes (Figure S8). [4]

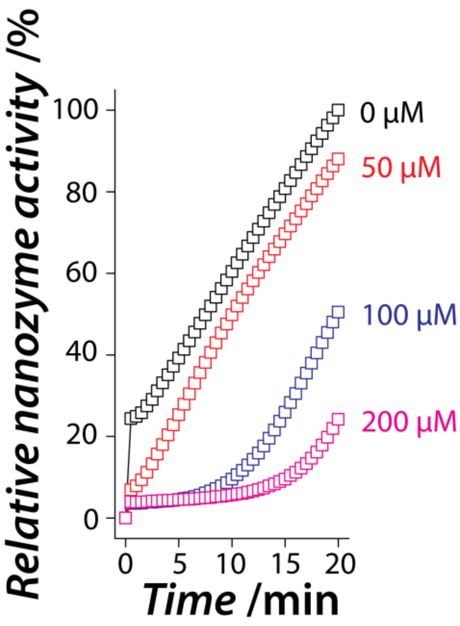

Figure S8. Change in the nanozyme activity of $Gd(OH)_3$ nanorods with increasing concentration of L-Cys as a function of time. Reprinted with permission from Langmuir 2017, 33, 38, 10006–10015. Copyright 2017 American Chemical Society.

In the current work, when the NiO nanozyme was exposed to L-cysteine molecule, the catalytic activity was permanently blocked (Figure 4). However, we observed that the extent of blocking of the catalytic activity was proportional to the concentration of L-cysteine. It is well-known that in natural enzymes, one inhibitor molecule inactivates a single enzyme molecule. Given that surface atoms of the nanozyme play the role of enzyme-equivalent active sites, it would require a high concentration of the inhibitor to completely inactivate a single nanozyme. If we think about the influence that this condition would have on the K_m and V_{max} of the nanozyme, we will see no change in the K_m. This is because irreversible binding of the L-cysteine to the nanozyme should not influence the binding of the chromogenic substrates to the surface of the nanozyme. On the other hand, given that there is a decrease in the overall surface atoms available for catalysis, we should observe a decrease in the V_{max} in the presence of L-cysteine. To understand if this hypothesis applies to our system, we calculated the K_m and V_{max} in the presence and absence of L-cysteine (Figure S9). In line with our hypothesis, we indeed observe a decrease in the V_{max} for both substrates as we increase the concentration of L-cysteine, while the K_m remains constant (Figure S9 and Table S1).

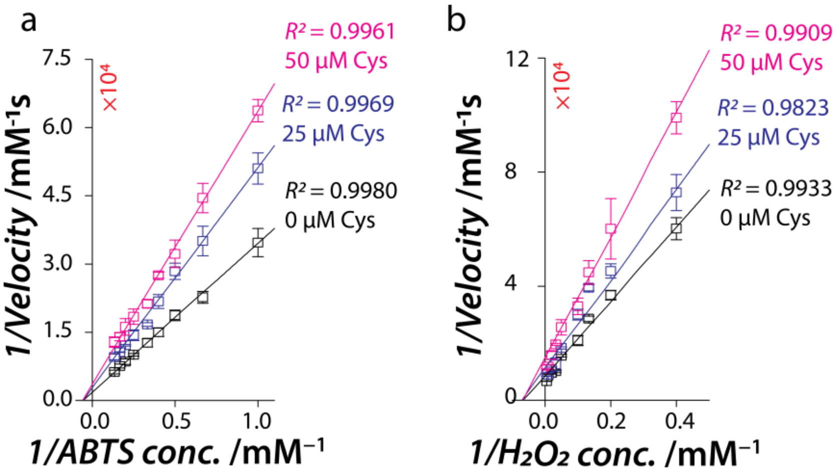

Figure S9. Lineweaver-Burk (LB) plots obtained for the NiO nanozyme in the presence of different concentrations of L-cysteine inhibitor. (**a**) shows the LB plots with varying ABTS concentration at a fixed 10 mM H_2O_2 concentration while (**b**)

shows the LB plots with varying H_2O_2 concentration at a fixed 0.5 mM ABTS concentration. The error bars represent the standard deviations obtained from three independent experiments. The linear fitting was performed using OriginPro 2016.

Table S1. Comparison of apparent enzyme kinetic parameters for the NiO nanozyme in absence and presence of different concentrations of L-cysteine.

	0 µM L-cysteine		25 µM L-cysteine		50 µM L-cysteine	
	**ABTS**	**H_2O_2**	**ABTS**	**H_2O_2**	**ABTS**	**H_2O_2**
K_m (mM)	17.5	14.8	17.5	14.6	17.3	14.8
V_{max} (mMs^{-1})	5.3×10^{-4}	1.1×10^{-4}	3.6×10^{-4}	9.1×10^{-5}	2.8×10^{-4}	6.90×10^{-5}

To confirm the binding of L-cysteine to the nanozyme, we also performed FTIR analysis of pristine L-cysteine, the nanozyme and a mixture of the nanozyme and L-cysteine. Figure S10 clearly indicates that the L-cysteine is bound to the surface of the nanozyme. This binding results in the inhibition of the catalytic activity.

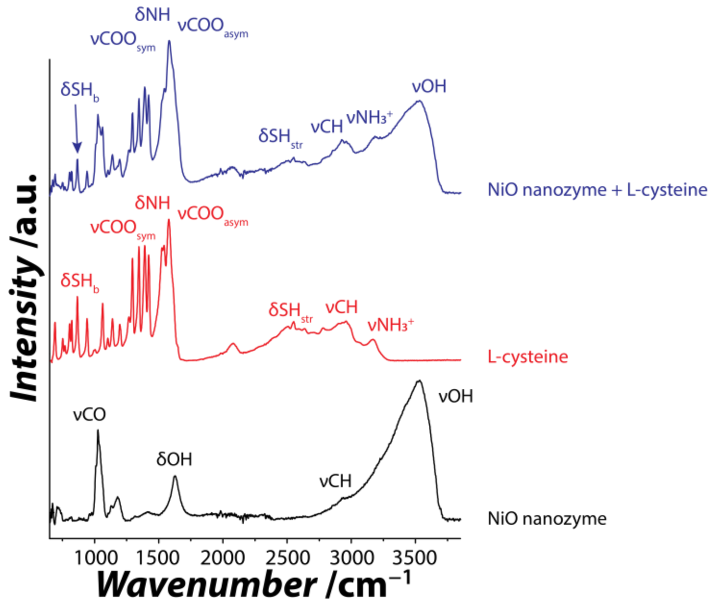

Figure S10. FTIR spectral analysis for NiO nanozyme (black), L-cysteine (red) and NiO and L-cysteine interaction (blue).

To confirm if the order of addition of the nanozyme, substrates and L-cysteine has any influence on the inhibition of the nanozyme activity, we performed the experiment in two ways:

(i) we first incubate the L-cysteine with the NiO nanozyme for 10 min before we add the two substrates (ABTS and H_2O_2) to the reaction

(ii) we first incubate the L-cysteine with H_2O_2 for 10 min, following which we add the ABTS and NiO nanozyme to the reaction

Further, the stability of the NiO nanozyme following its interaction with L-cysteine was assessed by performing XRD analysis (Figure S11). We observe no change in the crystal structure of the NiO nanozyme suggesting that the material remains stable.

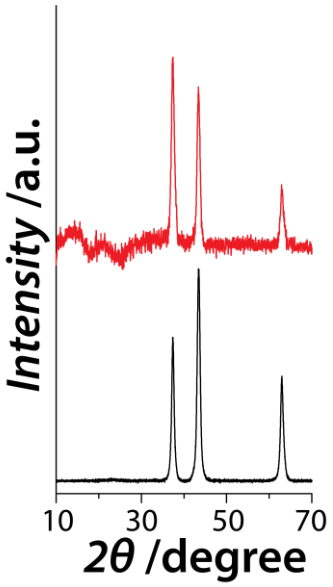

Figure S11. XRD spectra obtained from NiO nanozyme before and after its exposure to L-cysteine.

As shown in Figures S12, it is clear that the inhibition of nanozyme activity only occurs when L-cysteine is allowed to first interact with the NiO nanozyme. In the case where we first incubate the L-cysteine with H2O2, we only observe a marginal decrease in the nanozyme activity. This is possibly be due to the oxidation of L-cysteine to cystine which then interacts with the NiO nanozyme and shows marginal inhibition of the nanozyme activity (as shown in Figure 5).

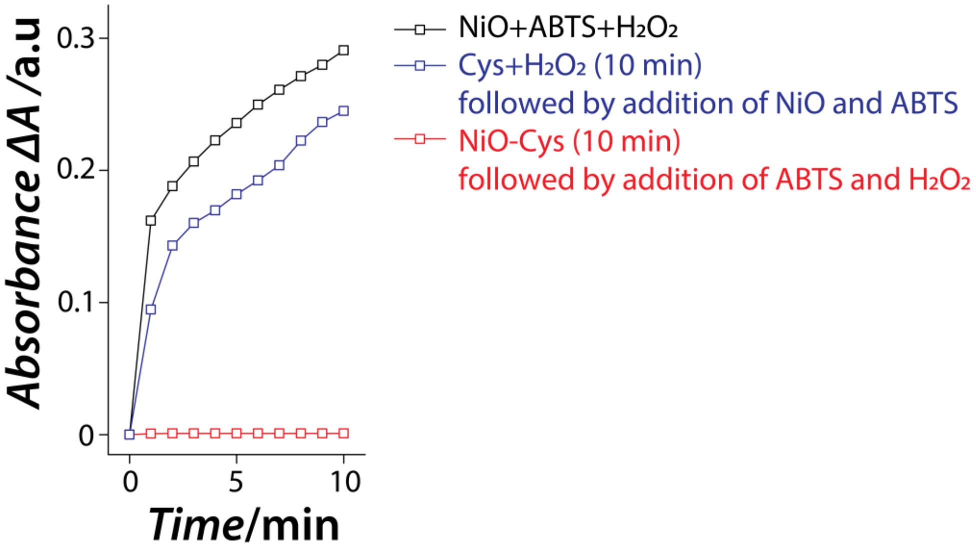

Figure S12. A plot of the change in absorbance as a function of time for (i) pristine NiO nanozyme exposed to ABTS and H_2O_2; (ii) L-cysteine exposed to H_2O_2 for 10 min followed by the addition of ABTS and NiO and NiO exposed to L-cysteine for 10 min followed by the addition of ABTS and H_2O_2.

References:

1. Wei, H.; Wang, E. Nanomaterials with enzyme-like characteristics (nanozymes): next-generation artificial enzymes. *Chem. Soc. Rev.* **2013**, *42*, 6060–6093, doi:10.1039/c3cs35486e.
2. Wang, X.; Hu, Y.; Wei, H. Nanozymes in bionanotechnology: from sensing to therapeutics and beyond. *Inorg. Chem. Front.* **2016**, *3*, 41–60, doi:10.1039/c5qi00240k.
3. Chen, W.; Li, S.; Wang, J.; Sun, K.; Si, Y. Metal and metal-oxide nanozymes: Bioenzymatic characteristics, catalytic mechanism, and eco-environmental applications. *Nanoscale* **2019**, *11*, 15783–15793.
4. Singh, M.; Weerathunge, P.; Liyanage, P.D.; Mayes, E.; Ramanathan, R.; Bansal, V. Competitive Inhibition of the Enzyme-Mimic Activity of Gd-Based Nanorods toward Highly Specific Colorimetric Sensing of l-Cysteine. *Langmuir* **2017**, *33*, 10006–10015, doi:10.1021/acs.langmuir.7b01926.
